# Patients’ and Health Care Professionals’ Experiences of a Digital Self-Management System for Asthma: Qualitative Study

**DOI:** 10.2196/79866

**Published:** 2026-03-20

**Authors:** Lovisa Jäderlund Hagstedt, Helena Hvitfeldt, Sara Riggare, Göran Petersson, Nadia Davoody, Maria Hägglund

**Affiliations:** 1 Department of Learning Informatics Management and Ethics, Health Informatics Center (HIC), Karolinska Institutet, Stockholm, Sweden; 2 R&D, Tiohundra AB, Norrtälje, Sweden; 3 Department of Women’s and Children’s Health, Participatory eHealth and Health Data, Uppsala University, Dag Hammarskjölds väg 14B, Uppsala, 75185, Sweden, 46 72 9999 381; 4 Centre for Disability Studies, Uppsala University, Uppsala, Sweden; 5 Department of Medicine and Optometry, Linnaeus University, Kalmar, Sweden; 6 Uppsala MedTech Science & Innovation, Uppsala University Hospital, Uppsala, Sweden

**Keywords:** asthma, digital health, empowerment, primary care, self-management, user experience

## Abstract

**Background:**

Living with asthma—especially in its severe forms—can significantly impact daily life, including social activities, work, travel, and household responsibilities. Collaboration between patients and health care professionals (HCPs) is frequently lacking, particularly regarding treatment goals. Self-management has been shown to mitigate the negative effects of asthma. Technical solutions might support self-management for patients with chronic diseases and their collaboration with HCPs.

**Objective:**

This explorative study aims to understand how patients and HCPs experience the use of a digital self-management system for asthma monitoring.

**Methods:**

This qualitative study was conducted at 5 primary care centers in Sweden and involved 20 participants: 14 patients who had utilized the digital self-management system Asthmatuner for at least 6 months and 6 specialist asthma nurses. Individual semistructured interviews were analyzed using qualitative content analysis to explore patterns and relationships within the data.

**Results:**

We identified 1 main theme, that is, “data-supported empowerment,” and 3 subthemes, that is, (1) empowerment by awareness, knowledge, and learning; (2) contact health care-patient; and (3) managing the monitoring. The theme of data-supported empowerment emerged as a synthesis of these findings, reflecting how the self-management system enabled patients to take a more active role in managing their medications and health. While most patients did not monitor their data continuously, they engaged with it when they felt it was necessary. Some patients expressed expectations of personalized follow-up from HCPs based on their monitoring data; however, these expectations were not always fulfilled. We also revealed a need to adapt and clarify the overlapping responsibilities of patients and HCPs.

**Conclusions:**

The digital self-management system for asthma was well received by both patients and HCPs, as it promoted empowerment. Clear communication about changes in workflow and responsibilities is essential to ensure the successful implementation of digital systems and improved health care delivery.

## Introduction

Health care faces multiple challenges, including an aging population, a growing number of individuals living with chronic diseases and multimorbidity, increasing demands from the public, limited resources, and expanding medical possibilities [1]. These challenges highlight the need for innovative approaches to health care delivery, particularly in primary care, where most chronic disease management takes place [2-4].

Person-centered care and patient empowerment are necessary to meet the needs of persons with chronic conditions [5-8]. The World Health Organization defines empowerment as “a process through which people gain greater control over decisions and actions affecting their health …” [9]. To achieve patient empowerment, the patient needs to understand his or her role, possess the necessary knowledge and skills, and be in a facilitating environment.

The concept of self-management is widely used in chronic care contexts and often refers to patients’ ability to manage symptoms, treatment, and lifestyle changes. Individuals with chronic conditions are often expected to take significant responsibility for their daily care and disease self-management [10]. However, this has also been critiqued for placing responsibility on individuals without sufficiently acknowledging structural, relational, and contextual factors that shape health behaviors [11,12]. While there is no universally accepted definition of self-management, this study adopts the definition: “… the individual’s ability to manage the symptoms, treatment, physical and psychosocial consequences, and lifestyle changes inherent in living with a chronic condition” [13]. Self-monitoring is a key component of self-management, as it enhances awareness and provides actionable information [10,14]. Effective self-management can improve health outcomes, reduce health care utilization, and lower overall health care costs [15-18]. In this study, we focus on the use of digital tools that support patients in their everyday self-management of asthma, while recognizing that such tools are embedded in broader care practices and relationships.

Digital health technologies have shown promise in supporting self-management of chronic conditions [19], with positive effects reported for both patients and health care professionals (HCPs) [20,21]. Asthma, a chronic respiratory disease, affects approximately 10% of the Swedish population, with higher prevalence among adult women [22,23]. Living with asthma—especially in its severe forms—can significantly impact daily life, including social activities, work, travel, and household responsibilities. Patients often report psychological distress and dissatisfaction with the continuity of care in primary settings. Moreover, collaboration between patients and HCPs is frequently lacking, particularly regarding treatment goals. These findings underscore the importance of structured patient education, which may be enhanced by digital health solutions [24].

In Sweden, asthma and chronic obstructive pulmonary disease nurses are registered nurses with several years of clinical experience and specialized training in respiratory care, including university-level courses in asthma, allergy, and chronic obstructive pulmonary disease. Their responsibilities encompass patient assessment, spirometry testing, education on disease management and inhalation techniques, smoking cessation support, and coordination of individualized care plans. These nurses play a central role in long-term follow-up and in supporting patients’ self-management [25].

Digital technology has the potential to improve both the health and care of patients with asthma [26-29], but there is limited evidence concerning patients’ experiences and their views of digital intervention in the self-management of asthma [28,30]. Customizable treatment plans and self-management through the self-monitoring of lung function and symptoms appear to be more effective than other forms of asthma self-management [31]. The digital tool AsthmaTuner (MediTuner AB) is a Conformité Européenne–marked self-management system that offers personalized treatment recommendations based on daily lung function and symptom monitoring [32].

The user experience of digital self-management interventions will have an impact on patients’ and HCPs’ adoption and continued use of such tools and is therefore important to understand. According to ISO 9241‐210, user experience is “a person’s perceptions and responses that result from the use or anticipated use of a product, system or service” [33] and includes emotions, beliefs, behaviors, and responses before, during, and after use, shaped by system functionality, prior experiences, and context.

This explorative study aims to understand how patients and HCPs experience the use of a digital self-management system (AsthmaTuner) for asthma monitoring.

## Methods

### Study Design

A qualitative approach was used to understand the experiences of patients and HCPs. The data were collected through semistructured interviews with patients and specialist nurses who had used the self-management system AsthmaTuner for at least 6 months. The COREQ (Consolidated Criteria for Reporting Qualitative Research) has guided the reporting of this study [34] (Checklist 1).

### Study Setting

The study took place at 5 primary care centers in Region Stockholm, Sweden. These centers are part of the primary care service in Tiohundra AB in Norrtälje. Norrtälje is the largest municipality area-wise in Region Stockholm; it is sparsely populated and has an aging population, and its inhabitants have lower socioeconomic status than other parts of Region Stockholm. This demographic and socioeconomic profile more closely resembles that of many municipalities across Sweden outside the major urban centers, making Norrtälje a relevant case for understanding primary care in rural and semirural contexts nationwide.

### The Self-Management System—Asthmatuner

**Figure 1.**
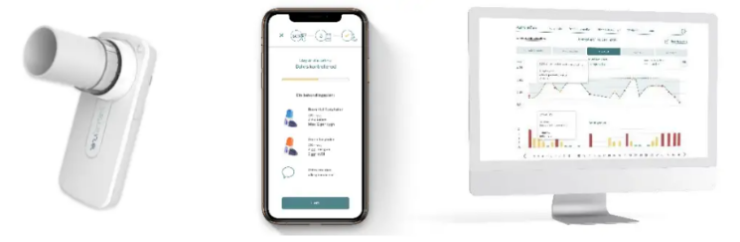
Overview of the components of the AsthmaTuner self-management system (wireless spirometer, patient app, and health care professional [HCP] interface).

Asthmatuner is a Conformité Européenne–marked self-management system for monitoring asthma. It consists of a portable wireless spirometer that can be used to measure lung function and a mobile app through which patients answer questions about their current asthma symptoms in conjunction with measuring their lung function. The app provides patients with a treatment plan based on their current symptom estimates and lung function. Figure 1 demonstrates an overview of the AsthmaTuner components. The treatment plan is presented in 3 levels—controlled, partly controlled, or uncontrolled—and suggests how the patient should take their medication. The app presents a view of trends in their symptoms and their lung function [32].

In addition, the self-management system includes an interface for the HCPs where patient data can be viewed and treatment plans prescribed. Being able to view patient data allows the HCPs to identify patients with poor asthma control in need of follow-up.

### Study Participants and Recruitment

Patients were recruited from the intervention arm of an ongoing randomized controlled trial in which 200 patients were given access to the self-management system, while the 200 patients in the control group were assigned to usual care and received a written or verbally communicated treatment plan. Written informed consent was obtained from all participants before inclusion.

Letters with information about the qualitative study were sent from the primary care centers to all patients who had used the self-management system for at least 6 months and were 18 years or older. Patients who responded to the invitation were subsequently contacted to schedule an interview. Invitation letters were sent out in 4 rounds as new patients became eligible. In total, 14 patients, 11 female/3 male, aged 44 to 76 years, mean age 64.5 (SD 10.1 y), participated in the study.

To ensure variation in the sampling, we applied a broad inclusion strategy to capture a wide range of experiences relevant to our study objectives. We used participant recruitment across different primary care centers from various geographic locations to reach a diverse audience, enhancing the representativeness of our sample. Despite a small sample, it corresponded roughly to the larger group regarding age and gender.

All specialist asthma nurses working at the participating primary care centers were approached and agreed to be interviewed (N=6). No further details (age or gender) about the nurses will be reported to ensure their privacy.

### Data Collection

Semistructured interview guides were designed by the research team and used for the interviews (MultimediaAppendices 1  2). Semistructured interviews were chosen to allow for both consistency across interviews and flexibility to explore participants’ individual experiences in depth. This method is well suited for qualitative research aiming to understand complex and personal phenomena, such as the use of a self-management system in health care settings [35]. The guides were based on previously constructed interview guides and modified using the team’s experiences of qualitative interviews. They were not pilot-tested due to time constraints. The interviews were conducted in Swedish either by phone or online, recorded, and transcribed verbatim. This approach allowed for greater flexibility in scheduling and reduced the need for travel or physical presence, which was particularly important given the participants’ varying health conditions, time constraints, and geographical locations. The interviews lasted 20 to 45 minutes, and no repeat interviews were performed. Only the interviewer and the participant were present during the interview. None of the interviewing researchers had any prior relationship with the patients, and they were not involved in patient care. The first author worked as a primary care physician at one of the participating care centers and, therefore, did not participate in data collection to avoid biased responses.

The first 10 patient interviews and all the nurses’ interviews were performed during 2021 to 2022. To reach data saturation from the patient interviews, 4 additional interviews were conducted in 2023. Since these interviews did not present any new findings, we concluded that saturation had been reached.

The participant checking of transcripts and analysis was not done to reduce the burden of participating in the study.

### Data Analysis

The interviews were analyzed using an inductive content analysis approach guided by Graneheim and Lundman [36].

**Figure 2.**
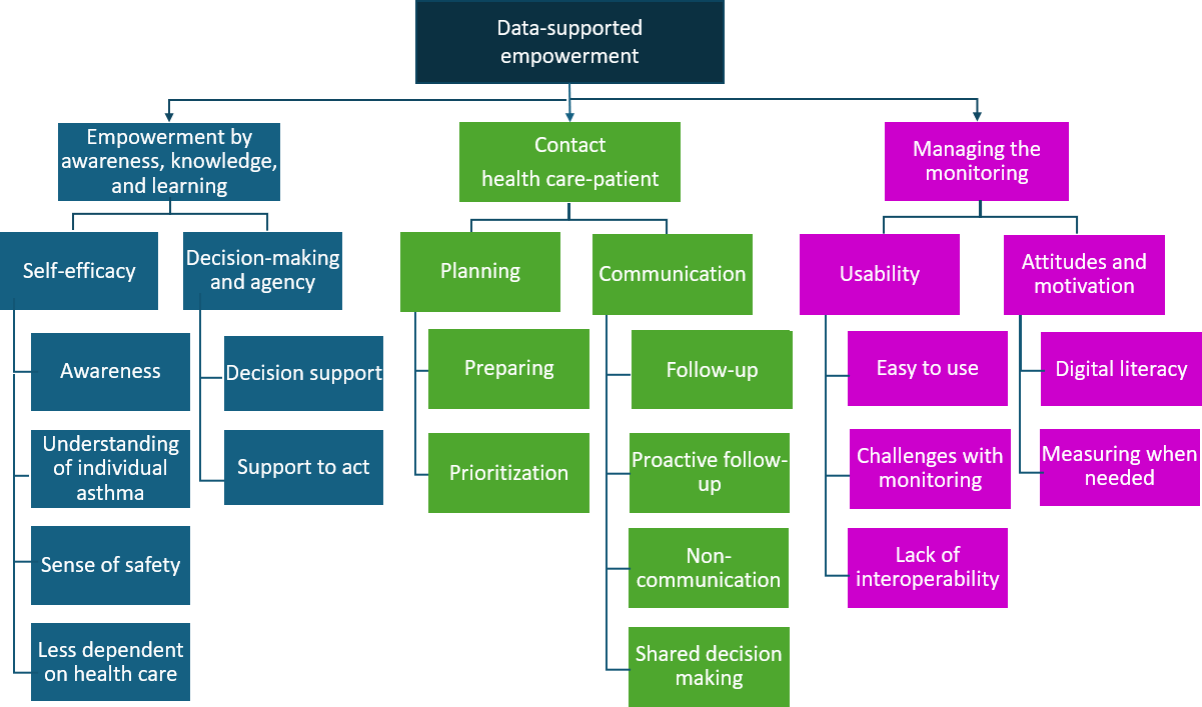
Main theme, subthemes, categories, and subcategories.

LJH, MH, ND, and SR performed the initial coding. They read all the transcribed interviews and shared the work of identifying relevant meaning units and the initial coding. To identify codes and subcategories, the coding process was first performed on the interviews with patients and nurses separately. ND, MH, and SR performed the initial coding of the patient interviews, and LJH and MH took the main responsibility for coding the nurses’ interviews. When subcategories had been identified, they were compared, and similarities and differences between the nurses and patients emerged. At this stage, the decision was made to continue the categorization and identification of subthemes jointly for both groups. This not only reduced the repetition of the findings due to similarities between groups but also enabled us to highlight the subcategories where nurses and patients expressed different experiences or opinions. To enhance transparency and support the trustworthiness of the analysis, we include an example of the coding process in line with the COREQ checklist [34] in Table 1. The complete results, with all themes and categories, are presented in the results section (Figure 2). The coding was structured according to the approach described by Graneheim and Lundman [36], where meaning units were condensed, coded, and grouped into subcategories and categories through an iterative interpretative process.

**Table 1.** Example of the analytical process, showing how meaning units were condensed, coded, and grouped into subcategories and categories, following the qualitative content analysis approach described by Graneheim and Lundman [36].

Subthemes of data-supported empowerment and their categories	Subcategory	Meaning unit
Empowerment by awareness, knowledge, and learning		
Decision-making and agency	Support to act	"I notice tendencies, like now it’s getting a little worse. And then I can start premedication before the values get too affected, so my asthma doesn’t have to become so bad.” [Patient 2, female, 58 years old]
Self-efficacy	Understanding of individual asthma	“I get an understanding of my asthma and when it gets worse. By measuring continuously, I know how I’m doing, and I can control my activities and my medication.” [Patient 14, female, 52 years old]
Contact health care-patient		
Communication	Proactive follow-up	“Sometimes they have called and said, ‘I keep ending up in the red zone. What’s wrong?’ They have contacted us then, just as they should when the device tells them to.” [Specialist nurse]
Communication	Noncommunication	“… I would have liked to have more feedback. But that doesn’t really have anything to do with the app.” [Patient 11, male, 71 years old]

Consensus regarding the categories and themes was reached through an iterative process in a dialogue between all four coders and HH, who also read all the interviews. The meaning units were translated into English after the analysis was completed.

### Ethical Considerations

The study received ethical approval by the Swedish Ethical Review Authority (2019‐01898). All study participants gave written informed consent. All transcribed interviews were pseudonymized before analysis, to ensure the participants privacy was preserved. Study participants were not reimbursed for their participation, in accordance with the ethical approval.

## Results

### Overview

The analysis resulted in the identification of 1 overarching theme: data-supported empowerment, which built on three subthemes: (1) empowerment by awareness, knowledge, and learning; (2) contact health care-patient; and (3) managing the monitoring. Each subtheme encompassed several categories relevant to both patients and HCPs, with some subcategories applying to only one of these groups (Figure 2).

Rather than aiming to identify a single main theme from the outset, the analysis explored patterns and relationships within the data. The theme of data-supported empowerment emerged as a synthesis of these findings, reflecting how the self-management system enabled patients to take a more active role in managing their medications and health. While most patients did not monitor their data continuously, they engaged with the data when they felt it was necessary. Some patients expressed expectations of personalized follow-up from HCPs based on their monitoring data; however, these expectations were not always fulfilled.

### Empowerment by Awareness, Knowledge, and Learning

#### Overview

The self-management system facilitated awareness, understanding, and knowledge of the patient’s condition and supported patients in taking a more active part in their care and treatment. Self-efficacy and decision-making and agency thus became the main categories of the subtheme empowerment by awareness, knowledge, and learning.

#### Self-Efficacy

The category self-efficacy refers to participants’ perceived ability to manage their asthma effectively, including interpreting symptoms, adjusting medication, and making informed decisions about daily activities. This interpretation is grounded in Bandura’s concept of self-efficacy, which emphasizes individuals’ belief in their capacity to influence outcomes through their actions [37]. Most patients experienced that monitoring gave them a better awareness of how asthma affects their well-being, and HCPs described how patients who used the self-management system became more interested in their condition and treatment.

For some patients, monitoring not only gave them a better awareness of asthma in general but also a higher level of understanding of their individual asthma.

*It gives me an understanding of my asthma and when it gets worse. By measuring continuously, I know how I’m doing, and I can control my activities and my medication.*

[Patient #14, female, 52 years old]

Patients who experienced this increase in awareness, which was also acknowledged by HCPs, used the monitoring to follow their lung function and adjust their treatment, and the monitoring also helped them to identify situations and factors that triggered exacerbations, such as different allergens or infections.

Several patients stated that the monitoring gave them a sense of safety because it helped them to determine whether their symptoms were caused by their asthma or something else, but also because they were encouraged to adjust their treatment if needed.

*I get good advice through it [the system]*. *It makes me feel safe. And my asthma has been great since I started using it*.

[Patient #4, female, 68 years old]

Some patients felt less dependent on health care as a result of using the self-management system since they could manage their asthma based on the treatment recommendations provided.

#### Decision-Making and Agency

The self-management system provided patients with information about their asthma control and suggested dosage adjustments of medications based on their current symptoms and lung function. Both patients and HCPs found that the self-management system served as an important decision support tool for the patients and gave them support to act on the suggestions.

*Yes, it feels like when I get bad measurements, I should increase [the dose of my medication]. Or it says I should take a bit more Bricanyl. I think it’s brilliant*.

[Patient #6, male, 61 years old]

*I notice tendencies, like now it’s getting a little worse. And then I can start premedication before the measurements get too affected, so my asthma doesn’t have to become so bad*.

[Patient #2, female, 58 years old]

### Contact Health Care-Patient

Interactions between patients and HCPs were affected by the self-management system, and 2 main categories were identified: planning and communication.

#### Planning

The self-management system was used by both patients and HCPs to prepare for planned follow-ups. Patients used it to recall variations in asthma control since the last visit, enabling them to give more informed answers.

HCPs used the data for prioritization when deciding which patients were most in need of active follow-up.

*This way, I get better control of how the patients with asthma are doing. And I can call the right patients; I can prioritize the patients who need to come here*.

[Specialist nurse #1]

#### Communication

Patient monitoring enabled digital follow-up. Patients and HCPs could discuss the data via phone or video consultations. The system was used for proactive follow-up, where nurses contacted patients with deteriorating asthma control and offered guidance. HCPs appreciated it when patients contacted them based on system alerts.

*Sometimes they’ve called and said, “I keep ending up in the red zone. What’s wrong?” They’ve contacted us then, just as they should when the device tells them to*.

[Specialist nurse #3]

However, there were also cases of noncommunication, where some patients experienced a lack of feedback from the HCPs, and some had problems contacting their specialist nurse.

During regular follow-ups, the data provided by the system were used for shared decision-making, where patients and HCPs examined and reflected on the collected data together.

*During a follow-up visit, you look at the registered data together with the patient, discuss how they have been, and try to find out what it was that made them deteriorate*.

[Specialist nurse #5]

### Managing the Monitoring

Usability and attitudes and motivation were the 2 main categories related to the management of monitoring.

#### Usability

Patients and HCPs agreed that the self-management system was easy to use. The HCPs noted that most patients did not have any problems using the system and were pleased to discover that older patients also managed to use the technology successfully.

*I am surprised at how many patients, even older people, can handle the digital technology, electronic identification, and an app like this*

[Specialist nurse #1]

Even though all patients thought the self-management system was easy to use, they also experienced some challenges with the monitoring. The most important challenge was remembering to use the device. Patients found that the option to use a reminder in the app was helpful, but after using it for some time, some patients got so used to the reminder that they no longer reacted to it.

HCPs did not report any challenges with monitoring, but instead a lack of interoperability, specifically concerning the need for the double documentation of the patient’s medications and treatment plan in the system’s care portal and the patient’s electronic health record.

#### Attitudes and Motivation

Digital literacy affects the patient’s attitude and motivation when deciding whether to try the self-management system. HCPs described how patients with high digital literacy were eager to try the self-management system, while those with low digital literacy were more reluctant and needed more support to make it work. For some patients, wanting to challenge themselves and learn how to use a new technology was a motivator.

*I thought, “this is going to be interesting. I must try this!” And then I also thought, as I often do, if you find that something is difficult, then you should definitely learn it.*

[Patient 8, female, 74 years old]

Patients were encouraged to use the self-management system at least once a week, although after using it for a while, most patients started measuring when needed, usually in connection with periods of exacerbation of their asthma. Some patients, however, planned to use it more regularly before a health care appointment to provide additional data on their current asthma control for the HCPs, indicating some variation in patients’ tracking strategies.

*I might not use it regularly, but when I start to feel that something is off, I immediately start using it, and then I can see, Yes, my asthma’s not good now. I’ll have to take more medication.*

[Patient #2, Female, 58 years old]

## Discussion

### Principal Findings

We found that the use of a digital self-management system for asthma was appreciated by patients and HCPs, as it supported empowerment and proved beneficial for both patients and HCPs. The system helped patients gain better knowledge about their chronic condition and provided them with tools and skills to manage the disease. The data generated by the system enabled HCPs to prioritize patients with greater needs for follow-up, thereby supporting a more efficient allocation of health care resources. By empowering patients who are willing and able to take greater responsibility for their own care, the system also allowed HCPs to dedicate more time to those requiring direct personal contact. However, self-monitoring is not without challenges. It demands additional effort and management from both patients and HCPs [38,39], highlighting the need to adapt existing health care delivery models to accommodate these new responsibilities.

The main theme, data-supported empowerment, was facilitated by patient-generated health data. This was particularly evident in 2 of the 3 subthemes: empowerment by awareness, knowledge, and learning, and contact health care-patient. The system helped patients learn about factors affecting their asthma symptoms, making them better equipped to manage their condition and proactively adjust their medications to prevent exacerbations. These findings are supported by previous research that has shown that digital technology can support patients in better understanding their symptoms and condition [30,40-43].

Consistent with earlier studies, the patient-generated health data provided by the system functioned as decision support for HCPs, helped patients become more informed, supported self-care, and contributed to shared decision-making [44,45]. These experiences align with Bandura’s definition of self-efficacy as the belief in one’s ability to organize and execute actions required to manage prospective situations [37].

Including both patients and HCPs in the study enabled the identification of differences in their experiences of using digital self-management tools. The subtheme Empowerment by awareness, knowledge, and learning was emphasized more by patients than by HCPs. However, HCPs appreciated the patients’ improved empowerment and self-efficacy and believed that this would positively affect the patients’ asthma control. Physicians have expressed similar views on patient-generated health data and have advocated for digital solutions to improve care [46].

Although patients can independently use the system, it may also create expectations that health care services cannot always meet. Some patients expected feedback from HCPs based on their reported data and were disappointed when this did not occur. However, some HCPs did proactively contact patients when they noticed a deterioration in asthma control. Personalized feedback is a key factor in achieving optimal outcomes and quality of care [41,47,48], but both patients and HCPs must have clear expectations and understand their respective responsibilities [49]. To ensure equity in care, it is also essential to establish routines for HCPs so that monitoring is streamlined and not dependent on the individual HCPs’ engagement.

In contrast to the findings from self-tracking in Parkinson disease, where patients found tracking burdensome and were unsure what to monitor [50], our study showed that patients were guided by the system and adapted their monitoring to periods when they experienced a greater need. Thus, the patients were not bound to monitor at certain times, but when they were motivated. This ad hoc pattern, rather than a continuous approach to self-monitoring, adopts a symptom-triggered, pragmatic strategy shaped by perceived relevance, burden, and confidence in their self-awareness. While this adaptive use may support sustainable engagement, it can also limit the early detection of gradual changes in asthma control. This needs to be considered in both the design of the self-management system, including in the development of algorithms to give treatment advice, and in the guidance to patients. The latter may include instructing patients to repeat measurements when they feel well on a regular basis, even if not measuring continuously, to ensure that the system does not interpret poor results as a new normal if patients only measure when experiencing worse symptoms. In illnesses where symptoms are not easily acknowledged, for example, diabetes or hypertension, the incentives to track may be experienced differently.

Certain findings in our study are specific to asthma, such as the importance of monitoring lung function and adjusting medication in response to symptoms. The system’s ability to support these tasks was central to participants’ positive experiences. At the same time, several themes, such as the need for tailored support, usability, and integration into daily routines, are likely relevant to other chronic conditions. By distinguishing asthma-specific needs from general principles of digital self-management, the study contributes both to condition-specific knowledge and broader digital health research.

This study shows that digital self-management systems, such as the one described in our study, may contribute to a novel way of delivering health care for individuals with chronic conditions, but it also highlights difficulties that must be addressed. Our findings support the potential of digital tools to facilitate self-management but also highlight the importance of considering the broader context in which such management occurs. As May et al [51] argue, self-management can impose a *burden of treatment*, particularly when health care systems shift responsibilities to patients without adequate support. This underscores the need to view self-management not only as an individual behavior but as a socially situated practice shaped by interactions with HCPs, access to resources, and systemic structures [52].

Well-functioning self-management systems have the potential to optimize care, save time for HCPs, and deliver more cost-effective health care. Further research is needed to determine whether digital self-management systems improve patient outcomes. The ongoing randomized controlled trial will provide additional insights into this. Future studies should also focus on establishing the factors necessary to implement and integrate self-management for patients, and its use by HCPs in primary care in order to maximize its gain.

The findings of this study suggest several implications for the design of digital self-management systems. For instance, patients valued features that provided immediate feedback on their asthma status, which enhanced their sense of control. However, some struggled with interpreting data, indicating a need for clearer visualizations and integrated guidance. Nurses emphasized the importance of seamless integration with existing systems to avoid the duplication of work. These insights point to the need for user-centered design that accommodates varying levels of digital literacy and supports both autonomous use and professional collaboration.

### Strengths and Limitations

In line with qualitative research traditions, particularly those using inductive content analysis, we formulated a broad aim rather than predefined research questions. This approach allows for openness to participants’ perspectives and supports the exploratory nature of the study, where themes and insights emerge from the data rather than being constrained by specific hypotheses [36]. The use of semistructured interviews also facilitated an in-depth exploration of participants’ experiences. Multiple researchers independently coded the data, enhancing credibility and reliability. Discrepancies were resolved through thorough discussions within the research team.

The data for this study were collected in 2021 to 2023. While digital self-management technologies continue to evolve rapidly, the key challenges identified by patients and HCPs, such as interoperability problems, varying levels of digital literacy, and their impact on attitude and motivation in using digital tools, remain highly relevant today. Nevertheless, there is a need for ongoing research to explore how digital health technologies may influence patients' and HCPs' experiences, engagement, and interactions with these tools.

A limitation of the study is the predominance of older patients (average age 65 years) and female patients. Age is a known barrier to adopting digital technology. However, participants were generally satisfied with the system’s usability, and younger users were unlikely to face additional challenges.

The interview guide was not pilot tested; however, it was carefully designed (by LJH, HH, MH), leveraging collective expertise and wide experience in the field. While it was neither formally validated nor pilot-tested, we ensured its robustness through several internal reviews and revisions. To facilitate participation among both patients and HCPs, interviews were conducted either online (via Zoom) or by telephone, based on the respondents’ preference. While this approach enabled flexibility and accessibility, it may have limited the ability to observe nonverbal cues and establish rapport in the same way as face-to-face interviews.

We consider the number of interviews with patients to have been sufficient to achieve saturation. That only 6 specialist asthma nurses participated is a limitation; however, this reflects the total number of eligible nurses at the participating units.

The relatively low number of patients who volunteered to be interviewed potentially creates a selection bias where those who did not respond to the invitation might have had a more negative perception of the self-management system. There is also a selection bias concerning which patients initially chose to try the self-management system; however, this would likely be replicated in a real implementation, making our results relevant to future users of the digital self-management system. Yet, further studies involving a broader and more diverse group of patients, including those who chose not to engage with the self-management system, are needed to better understand barriers to adoption and to capture a wider range of experiences.

While the qualitative nature of the study does not guarantee the generalizability of the results, we argue that they are transferable to similar patient groups and contexts. Still, we cannot exclude that some patient groups cannot take advantage of the self-management system. To counteract that, HCPs must be observant and identify those and provide physical contact when needed.

### Conclusions

This study provides novel insights into the real-world use of a digital self-management system for asthma, drawing on the perspectives of both patients and specialist nurses after extended use. A key contribution of this study is the identification of how digital self-management systems can both empower users and simultaneously shift responsibilities in ways that require careful negotiation between patients and HCPs. These findings underscore the importance of clear communication about expectations, roles, and responsibilities when implementing digital tools in chronic care.

Moreover, the study emphasizes the need for systems to be adaptable to individual needs and seamlessly integrated with existing digital infrastructures to support sustainable use. Patients and HCPs must know how to interact with and what to expect from these systems for them to promote a better way of delivering health care for people with chronic conditions. Clear communication about changes in workflow and responsibilities is essential to ensure successful implementation and improved health care delivery.

By focusing on asthma, a condition with specific self-monitoring and treatment demands, this study adds to the understanding of how digital tools can be tailored to support condition-specific needs while also offering transferable lessons for the broader field of chronic disease management.

## Supplementary material

10.2196/79866 Multimedia Appendix 1 Interview guide health care professionals.

10.2196/79866 Multimedia Appendix 2 Interview guide patients.

10.2196/79866 Checklist 1 COREQ checklist.

## Abbreviations

COREQ: Criteria for Reporting Qualitative Research
HCP: health care professional
