# Supplementary material for: Patients’ and Health Care Professionals’ Experiences of a Digital Self-Management System for Asthma: Qualitative Study
Source: JMIR Hum Factors. 2026 Mar 20;13:e79866. doi: 10.2196/79866 (PMC13004591; doi:10.2196/79866)
Supplement: Multimedia Appendix 1 [file humanfactors-v13-e79866-s001.docx]

Interview guide for healthcare professionals

**Introduction to the interview**

1. Gender (does not need to be asked but more noted)
2. Age
3. Occupation/profession
4. Years in the profession
5. How long have you worked with Asthma/COPD patients?
6. How long have you been using Asthmatuner in your work?
   1. How do you experience the introduction of “Asthmatuner”?
   2. What was positive? What was negative? Give examples.

[*implementation process, communication from management, training, technical support, etc.*]

1. Have you experienced any difficulties/challenges with the introduction of "Asthmatuner"? Give examples!
2. What benefits/gains have you experienced with the introduction of "Asthmatuner"? Give examples!
3. Can you describe how you use "Asthmatuner" in your work?

**About specific patient visits/patient encounters**

1. Do your patients specifically refer to “Asthmatuner” during visits/patient appointments?
   1. If yes –
      1. In which cases does this usually happen?
      2. Do you look at information from “Asthmatuner” together?
      3. Do you have to explain how to use the system or how to interpret information from the system?
      4. Do they ask questions based on information from “Asthmatuner”?
2. Is the visit itself affected by the patient using “Asthmatuner”, e.g. content such as discussion topics or time spent?

**Overall effects of patient use of “Asthmatuner”**

1. Have you noticed any impact on the quality of care?
   1. If yes, give examples!
2. Do you feel that the patients' use of “Asthmatuner” has affected their compliance with prescribed treatment?
3. Do you feel that patients who use “Asthmatuner” are supported in their self-care, e.g. easier to adjust medication as needed?
4. Do you feel that “Asthmatuner” has influenced patients' knowledge about their asthma?

**Impact on work environment and tasks**

1. In your experience; has the introduction of “Asthmatuner” affected your work?
   1. If yes –
      1. In what way?
      2. Has your workload been affected? How has it been affected?

[more/less admin? more/less questions? phone calls?]

- - 1. Has the work process been affected? How has it been affected?
    2. Have your interactions with other healthcare professionals been affected?

1. Do you feel that “Asthmatuner” is well integrated with your other digital work environment?
   1. Do you need to double document any information in “Asthmatuner” and in other IT systems?
   2. Do you need to log in separately to “Asthmatuner”?
2. Has the way you prepare for a patient visit been affected by “Asthmatuner”? Is there a difference in preparations between a visit with a patient who uses “Asthmatuner” and a patient who does not?
   1. If yes - in what way?

**Concluding questions**

1. Do you have any suggestions on how “Asthmatuner” could be improved to be more useful for you and your patients?
2. Is there anything we have missed to ask you about? Anything you would like us to know about using “Asthmatuner”?
