# Supplementary material for: Patients’ and Health Care Professionals’ Experiences of a Digital Self-Management System for Asthma: Qualitative Study
Source: JMIR Hum Factors. 2026 Mar 20;13:e79866. doi: 10.2196/79866 (PMC13004591; doi:10.2196/79866)
Supplement: Multimedia Appendix 2 [file humanfactors-v13-e79866-s002.docx]

Interview guide patients

**Introduction to the interview**

1. Gender (does not need to be asked but more noted)

2. Age

3. How long have you had Asthma?

a. Can you tell us a little about your medical history? Have you had many problems with asthma? Acute episodes of care?

4. Why did you choose to join the study?

[*Interested in new technologies, digital services, difficulties to manage your disease, contribution to research, healthcare professionals suggested it, good to have more frequent follow-up*]

**About your use of "Asthmatuner"**

5. How long have you been using “Asthmatuner”?

a. How did you experience starting to use “Asthmatuner”? How were you

introduced to the technology?

b. What has been positive? What has been negative? Examples...

[*implementation process, communication and information from health professionals, training, technical support, etc.*]

6. What were your expectations before you started using "Asthmatuner"?

7. Can you describe how you use "Asthmatuner"?

[*What do you do? How often?*]

8. What do you think about the usability of "Asthmatuner"? Do you understand how it works and how to use it?

9. Have you experienced any difficulties/challenges since you started using "Asthmatuner"? Give examples!

10. Have you experienced any benefits/gains since you started using "Asthmatuner"? Give examples!

11. Do you want to continue using "Asthmatuner" after the study is over?

[*Why/why not*]

**When you visit the healthcare center**

12. How has the use of “Asthmatuner” affected your relationship with healthcare?

a. improved/deteriorated communication?

b. more/less number of visits?

c. increased/decreased trust in healthcare?

d. worsened/improved your health?

e. motivated you to make lifestyle changes?

f. opportunity to make decisions about treatment and care?

13. Do you discuss "Asthmatuner" with your healthcare professionals?

a. If yes -

i. In which cases does this usually happen?

ii. Do you look at information from “Asthmatuner” together?

iii. Do health professionals ask questions based on information from “Asthmatuner”?

b. In what way do you experience that your healthcare professionals support you in using services like "Asthmatuner" and/or other digital services?

c. Does the use of “Asthmatuner” affect the healthcare visit itself, e.g. content such as discussion topics or time spent?

**Effects of using "Asthmatuner"**

14. Do you feel that your use of “Asthmatuner” has affected your adherence to prescribed treatment?

15. Do you feel that your use of “Asthmatuner” supports you in your self-care, e.g. easier to adjust medication as needed?

16. Do you feel that “Asthmatuner” has affected your knowledge about your asthma?

[*general knowledge about asthma/knowledge about how you function and react*]

17. Does "Asthmatuner" support you in making informed decisions about your health?

a. If yes, in what way?

18. Does “Asthmatuner” affect how confident you feel that you can manage your asthma well?

19. Has “Asthmatuner” affected the way you prepare for a healthcare appointment? Do you do anything differently today?

a. If yes - in what way?

**Managing your health information and digital services in general**

20. Do you use any other health-related digital services/tools?

[*1177 Healthcare Guide, health apps*]

21. How do you manage your health information? E.g. information about your healthcare visits, information about your illness and health (saves, prints paper copies of all documents; keeps information on computer; on mobile phone; has everything in one folder; documents and information, e.g. links, articles, books in different places)

22. Do you otherwise use the Internet to find information about your health?

a. What sources do you use to look for health information?

**Concluding questions**

23. Would you recommend other people with asthma to use "Asthmatuner"?

24. Do you have any suggestions on how ”Asthmatuner” could be improved to be more useful?

25. Is there anything we have missed to ask you about? Anything you would like us to know about using “Asthmatuner”?
